# Seeking HELP beyond the pill: Women’s perceptions of informed consent for medication abortion: Mixed methods research

**DOI:** 10.1371/journal.pone.0349065

**Published:** 2026-06-10

**Authors:** Maka Tsulukidze, Gabriele Di Cicco, Tessa Longbons Cox, Katherine A. Rafferty, Stuart W. Grande, Ingrid Skop, David Collison

**Affiliations:** 1 Marieb College of Health and Human Services, Florida Gulf Coast University, Fort Myers, Florida, United States of America; 2 Charlotte Lozier Institute, Arlington, Virginia, United States of America; 3 Centre for Social Cognitive Studies, Institute of Psychology, Jagiellonian University, Krakow, Poland; 4 Communication Studies Program, Department of Psychology, Iowa State University, Ames, Iowa, United States of America; 5 Public Health Administration & Policy, University of Minnesota School of Public Health, Minneapolis, Minnesota, United States of America; University of Foggia: Universita degli Studi di Foggia, ITALY

## Abstract

Informed consent is a cornerstone of patient autonomy, yet its implementation in medication abortion, especially in the context of rapidly expanding telehealth and online access, remains understudied. This study explores how informed consent is experienced by women who undergo medication abortion, focusing on the information they receive and the gaps they perceive. Using an exploratory sequential mixed methods design, we first conducted a thematic analysis of online narratives to examine how women described their experiences with information, support, and uncertainty. We then developed and administered a national survey informed by these findings to assess how women perceive the informed consent process. Thematic analysis of online narratives revealed four key patterns: women sought information about medication abortion symptoms and side effects; emotional support; urgent reassurance during the process; and clarification of the information provided. Survey results showed that concerns about fetal remains, emotional well-being, and the risk of an incomplete abortion (retained tissue) were strongly associated with women’s sense of being informed. Emotional responses also shaped these perceptions, where women who felt stressed were less likely to feel adequately informed, while those women who felt happy were more likely to report receiving sufficient information. Integration of qualitative and quantitative findings revealed consistent concerns, particularly about unanticipated symptoms, emotional distress, and the need for clearer guidance. Our results suggest that some women experience uncertainty or unmet informational needs prior to taking abortion medications, regardless of care setting. This informational gap raises the possibility of preference misalignment, whereby patients’ expectations may not fully reflect their lived experience. The findings highlight a pressing need for clearer, more comprehensive, and emotionally supportive consent practices, particularly as medication abortion becomes increasingly accessible and utilized by women. Addressing these gaps can strengthen patient-centered care and ensure that women’s choices are informed, respected, and aligned with their values.

## Introduction

Informed consent, regarded as a fundamental principal right in health care, is a patient’s capacity to assess and make decisions regarding their medical care [1]. The American Medical Association’s Principles of Medical Ethics defines informed consent as occurring “when communication between a patient and physician results in the patient’s authorization or agreement to undergo a specific medical intervention.” According to the AMA principles, doctors must determine the patient’s capacity to understand medical information and alternatives and make an independent decision, while also providing the patient with accurate information in accordance with the patient’s communication preferences. Doctors must provide the patient with information about the diagnosis, recommended treatment(s), and the risks and benefits of all options [2]. Hence, in contemporary bioethics and in clinical practice, informed consent is regarded as a dynamic communicative process between patients and providers that moves beyond a single transaction or signed form. However, empirical work shows that standard consent practices often produce limited understanding for patients and treat informed consent as a medico‑legal requirement, thus prompting calls to reconceptualize consent as an ongoing patient‑centered dialogue that supports deliberation and shared decision‑making over time [3–9].

Informed consent plays a critical role in reproductive health and women’s experiences with the medical care they receive during an abortion. A communication-based approach to informed consent is especially salient for medication abortion in telemedicine settings, where informational needs, emotional responses, and opportunities for clarification may evolve across the course of care. The National Abortion Federation (NAF) clinical policy guidelines emphasize that both informed consent and patient counseling are required for patient decision-making during the abortion informed consent process [10]. According to the NAF informed consent standards, the patient must be given information about the abortion procedure, available alternatives, and the opportunity to ask questions. The provider must document that the patient is fully informed, has made her decision voluntarily, and has been properly counseled regarding risks and benefits associated with the type of procedure she has elected, including the risk of hemorrhage, infection, organ damage, continuing pregnancy, and possible death [10]. A survey of abortion patients in the United Kingdom found that feeling well-informed and having alignment between the information provided and the actual abortion experience were both associated with perceptions of high-quality care [11]. For women choosing medication abortion, it is particularly salient to communicate potential risks and side effects, explain how to respond to emergencies, and remain attentive to patients’ emotional needs [12]. However, scant research has examined what information women seeking medication abortion need to feel fully informed.

The COVID-19 pandemic drastically changed women’s abortion experience and the informed consent process and women’s information seeking behaviors. Notably, women requested abortions earlier in their pregnancy and more women used telemedicine and teleconsultations for abortion support [13]. The expansion of telemedicine abortion since 2020, coupled with the wide variance across state-level abortion policies has led to significant differences in how medication abortion is provided across the U.S.

There is conflicting evidence on women’s experiences with tele-abortion. Some research indicates that many women are satisfied with telemedicine abortion and that both synchronous and asynchronous care are acceptable, effective, and empowering for women [14,15]. A qualitative study of 30 women undergoing medication abortions found that both telemedicine and in-clinic patients felt interpersonally connected to their providers [16]. Moreover, telemedicine abortion has been described as a “high quality, person-centered care model that can empower patients” [17]. In contrast, other research suggests that some women who choose medication abortion experience tensions surrounding their decisions and report feeling misinformed or unprepared, even after discussing the process with their abortion provider [18–22]. Furthermore, several complications of women using tele-abortion were recorded post-COVID, noting women’s increased pain, lack of psychological support, bleeding, and the need for blood transfusions [13].

As medication abortion comprises a growing majority of all abortions in the United States [23], more research analyzing the information needs of women undergoing medication abortion both online and in-clinic, and whether those needs are fully met, is warranted. Thus, we studied women’s perceptions of the information received as part of the informed consent process for their medication abortion.

## Materials and methods

**Fig 1:**
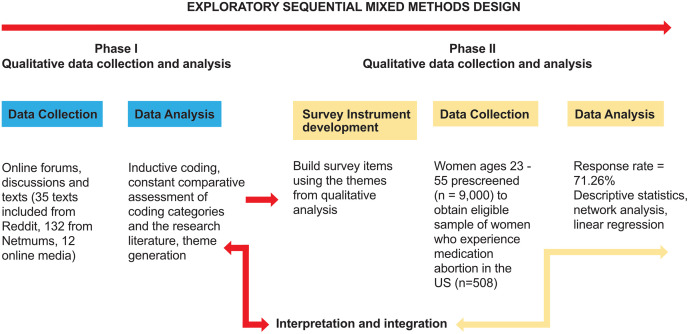
Visual model of the research design.

We used an exploratory sequential mixed methods design to ensure that the perspectives and experiences of women who have undergone medication abortion meaningfully shaped the development of our quantitative instrument. This approach allowed us to first gain in-depth insights into women’s perceptions and unmet needs related to informed consent through qualitative analysis of personal narratives (Phase I). These findings then informed the development of a targeted survey (Phase II) to assess the prevalence and variation of these experiences across the study sample. This design was well-suited to our aim of both exploring complex, nuanced experiences and generating generalizable data to inform improvements in informed consent practices for medication abortion. A visualization of the research design is provided in Fig 1.

Therefore**, Phase I** centered on understanding women’s experiences with medication abortion and the information and support they felt they needed. **Phase II** applied the knowledge gained in Phase I to develop an online survey assessing 362 women’s experiences with informed consent during their medication abortion. Recruitment started 02/09/2024 and ended 05/06/2024. All participants were provided with informed consent as approved by the Florida Gulf Coast University (FGCU) Institutional Review Board (IRB). Consent was obtained electronically in writing via the Qualtrics survey platform. Participants were presented with the consent information at the beginning of the survey and were able to choose whether to continue the study. Only those women who agreed to participate proceeded with the survey; those women who declined were able to exit the survey and received a thank-you message.

### Ethical considerations

The research protocol was reviewed and approved by the Florida Gulf Coast University Institutional Review Board.

In the qualitative phase of this study, all data were obtained from publicly accessible online forums and media publications. Data collection and analysis were conducted in accordance with the terms and conditions of the respective platforms, including Reddit and Netmums. No data were collected from private, password-protected, or restricted sources, and no interaction with forum users occurred. The authors did not receive any special access privileges to these data sources beyond what is publicly available or contractually permitted through standard platform use. Since the data were obtained solely from online public discussion forums and chats, no informed consent was required.

In the quantitative phase, survey participants were recruited through Prolific, and all recruitment, compensation, and data collection procedures complied with Prolific’s terms of service. The survey was administered via Qualtrics in accordance with its platform policies. Prolific applied our specified inclusion criteria to identify eligible individuals and recruit participants. Potential participants were presented with a link to the survey hosted on Qualtrics, where they could review the study information before deciding whether to participate. Consent was obtained electronically within Qualtrics, and participants indicated agreement prior to beginning the survey. Participation was entirely voluntary, and individuals could decline by simply exiting the link. Compensation for survey completion was managed directly through Prolific’s platform; funds were deposited into our account and Prolific distributed payments to participants. We were not directly involved in the payment process at any point.

As researchers, we also considered the inclusion of Internet-based data. A growing body of research has begun to critically examine the ethical complexities of using online content for research from platforms such as Reddit, raising important questions about participant anonymity, informed consent, and the blurred lines between public and private digital spaces [24–26]. While some scholars recommend removing usernames or handles to protect user identities, we recognize that such steps do not necessarily prevent re-identification of sources as direct quotes can still be traced to their original sources through search engines, potentially undermining anonymization efforts.

Nevertheless, we argue that uncensored, first-person accounts hold exceptional value, particularly when researching sensitive and stigmatized topics like abortion. These narratives offer unfiltered, nuanced insights into patient experiences that are difficult to access through traditional methods. Preserving the authenticity of these voices is not only methodologically valuable but also ethically defensible, especially when approached with care and in line with established guidelines such as those issued by the Association of Internet Researchers [27]. Additionally, in accordance with ethical guidance for research using publicly available internet data, all direct identifiers associated with online posts, including usernames, handles, and timestamps, were removed from quoted material in the manuscript. Only information necessary for analytic interpretation was retained. This approach was taken to minimize the risk of re-identification while preserving the substantive content and authenticity of first-hand narratives.

### Data access and availability

The quantitative survey data included sensitive information related to reproductive health experiences, emotional responses to abortion and perceptions of informed consent. Although the data were de-identified for analysis, public sharing of the dataset poses a risk of potential re-identification and participant harm due to the sensitive nature of the topic. As a result, public release of the full dataset is restricted in accordance with the approved research protocol. These restrictions were imposed by the Florida Gulf Coast University Institutional Review Board. De-identified survey data may be made available upon reasonable request for the purposes of replication or secondary analysis, subject to IRB approval.

### Phase I

#### Data collection.

To build our understanding of women’s experiences and information-seeking behaviors related to medication abortion, we applied an initial systematic search using Google to identify medication abortion-related online platforms. The search strategy included variations of keywords combining abortion (e.g., medical abortion, medication abortion, mifepristone, misoprostol, drugs), consultation (e.g., encounter, visit), consent (e.g., risks, benefits), and pregnancy (i.e., full search strategy will be available upon request). Searches were independently conducted by three researchers (MT, SG, TLC), and results were saved as time-stamped PDF files. Next, all relevant websites were accessed and screened by the same three researchers (MT, SG, TLC) for meeting the following inclusion criteria: 1. Containing women’s personal experiences about their medication abortion; and 2. Containing discussions written in the English language. To focus exclusively on women’s direct experiences with medication abortion, we excluded websites that provided information from federal, or state agencies intended for legal or educational purposes, as well as content from explicitly pro-choice or pro-life organizations, news reports on legislative developments, and accounts from women who had abortions outside the U.S.

#### Qualitative data source.

It is important to note that individuals who share experiences or seek information in online forums represent a self-selected subgroup. Engagement in online information-seeking does not necessarily indicate deficiencies in clinical counseling, but may reflect a range of behaviors, including reassurance-seeking, cross-checking information, managing uncertainty, or a preference for multiple sources.

Our initial online searches produced 2,331 documents. Of these, the majority were articles about medication abortion that did not include women’s own perspectives, and only 14 documents met all the inclusion criteria. Based upon the results of this initial search strategy, we conducted an expanded search of two forum websites, Netmums and Reddit, removing any duplicates identified during the initial search. These forums were selected for expanded inclusion due to being the most relevant and data-rich online sources. In addition, these websites are not openly politicized, women post anonymously, and these platforms are a space for unsolicited stories with no reward or compensation to those who post. Although Netmums is based in the United Kingdom, we determined it met our inclusion criteria after confirming with the website administrator that U.S. women also use the website to share their experiences. In addition, we excluded any discussion posts that contained a reference to the UK health system (e.g., NHS, GP, midwife, BPAS, A&E) and any linguistic nuance indicating UK characteristics (e.g., ring/phone a hospital, gynae (short for gynecologist), flat, UK format of date and month notation, e.g., 19th June).

Discussion threads regarding medication abortion on Netmums contained data from a total of 15 pages with 30 threads on each page, except page 15, which included only six threads. Of these, 199 threads were screened for inclusion, with 132 posts included in the final dataset after excluding explicitly non-US based posts. Because Reddit is a large website with many forums, or “subreddits,” we used Google to conduct a search of the whole site to draw posts from multiple subreddits. We reviewed the first 33 pages of results, which produced 322 threads that were screened for inclusion. Thirty-five were included in the final dataset.

We also included 12 online texts with stories about medication abortion reported by media outlets such as *Today*, *Vice*, and *Cosmopolitan*. While these third-party reports differed from the firsthand narratives shared in online forums, they often provided additional details, particularly about the informed consent process, that were missing or only briefly mentioned in women’s personal stories. These sources helped fill important gaps in the forum narratives, offering greater substance around what information was conveyed, how it was communicated, how women interpreted their experiences, and larger social discourses surrounding informed consent.

Our final dataset included posts from 2016–2023, reflecting a wide range of experiences with medication abortion. Most posts were published in 2020–2023.

#### Data analysis.

We applied an inductive coding process, which was suitable for analyzing raw, unedited, and uncensored online forum data [28]. Simultaneously, we used constant comparative methods [29] to assess differences and similarities between our codes of the discussions and the research literature on informed consent. The purpose of performing this comparison was to generate broader concepts from the data and to help explain women’s experiences seeking information regarding their medication abortion.

The iterative process of data collection, data analysis, and development of coding categories, allowed for initial codes to develop into inductive themes. The identification of themes was based upon frequency, extensiveness, and intensity of different codes [30]. To ensure the clarity and accuracy of the themes, the initial list of themes developed by two coders (MT, SG) was reviewed by two additional researchers (KR and IS). The finalized list of themes was agreed upon by all four coders, and the conceptualization of each theme was further developed during coding meetings. The data reduction process of moving from initial codes to more refined codes and concrete themes continued until saturation was achieved [28]. Throughout the entire data analysis process, researchers made all decisions collaboratively through regular peer debriefing meetings and creating written memos. Any ambiguity was resolved through group consensus.

#### Trustworthiness and rigor.

Trustworthiness assesses the credibility, transferability, dependability, and confirmability of the data collection and analysis processes [31]. We upheld these principles by beginning with a careful design that clearly defined its purpose, research questions, and notion of “boundedness” (i.e., establishing the limits and context of the case) [32]. Concrete decisions surrounding inclusion and exclusion criteria were established and agreed upon by the two coders involved in the data collection and initial coding process. Two additional coders were brought in later to the data analysis process to review the data and ensure the clarity and accuracy of themes. Sufficient time was spent on the entire data collection and analysis process, transpiring over the course of 15 months. Principles of reflexivity were used throughout the inductive coding process, with the writing of individual and group memos that provided a transparent data audit [31,32]. Finally, we had a team of three female coders and one male coder, which allowed for the presence of multiple perspectives to be reflected and discussed.

### Phase II

#### Recruitment.

The survey was generated using Qualtrics software, (Qualtrics, Provo, UT, version: February 2024). The participants were recruited via Prolific [February – March 2024] [33] using prespecified eligibility criteria; the resulting sample constitutes a nonprobability online panel. Survey eligibility criteria included: women between 23–55 years, obtained medication abortion in the U.S., and English language comprehension. To obtain an eligible sample, we pre-screened 9,000 women. The final sample included 508 women, of whom 362 responded to the survey.

#### Survey development and operationalization of qualitative themes.

Findings from Phase I directly informed the development of the Phase II survey instrument. Specifically, themes identified through thematic analysis were translated into measurable survey items to assess the prevalence and salience of information needs and emotional responses observed in the qualitative data (Table 1). For example, the theme of *seeking information about abortion symptoms and side effects* informed survey items assessing concerns about bleeding, pain, retained tissue, and failed abortion. The theme of *seeking emotional support* informed items measuring emotional responses such as stress, relief, happiness, guilt, and fear, as well as concerns related to mental and emotional health. Themes related to *urgent reassurance and discussion of information provided* guided the inclusion of items assessing perceptions of preparedness, clarity of information, and overall sense of being informed prior to taking abortion medications.

**Table 1:** Joint display for survey instrument development: Integrated item generation matrix.

Qualitative themes and sub-themes	Survey items	Rationale for the question
**Seeking information and advice on abortion symptoms and side effects**	Did you desire to receive more information about the medication abortion process than what was offered? [If yes, please explain what additional information you would have liked to receive.] What, if any, complication(s) or issues after your abortion did you most feel you needed information about? What types of physical complications did you experience? Please check all that apply.	Elicit women’s perception about the need /desire to receive information
**Seeking help and support for emotional response to abortion**	What types of emotions did you experience after having the abortion? Please check all that apply even if they seem contradictory	Collect information on the types of emotions experienced by the women
**Seeking urgent support and reassurance about medical abortion**	What was your main source of information for preparing yourself for the abortion?	Identify the sources of information available to women as they undertook medication abortion
**Discussing information provision as part of the medication abortion process**	Perceptions about communication with medication abortion provider. Perceptions about the received information [whether information was perceived as comprehensive, explained risks and benefits, all available options including surgical abortion, contact information for emergency/complication, information about possible complications]	Elicit women’s perception about the readiness to manage any abortion complications, and perception of being informed and ease of communication with the provider to receive necessary information

Survey items were reviewed by the research team to ensure conceptual alignment with qualitative themes and clarity of interpretation. This exploratory operationalization approach is consistent with best practices for sequential mixed methods research, in which qualitative findings are used to inform quantitative measurement [34,35].

After the items were developed, we solicited feedback from subject matter experts, including women who have had abortions or who have provided support to women who had abortions, to refine the survey instrument.

### Statistical analysis

The questionnaire gathered participants’ demographic background and abortion experiences through several measures. These included multiple selections related to the abortion process, such as the provider, the U.S. state where the abortion occurred, and the details of the informed consent they received prior to their medication abortion—whether and how it was received and given, who was involved in the process, and whether it included information about possible complications. Participants also indicated if they desired more information about the medication abortion process and identified the issues on which they desired more information (e.g., bleeding, failed or incomplete abortion) and their main sources of information (e.g., abortion provider, Internet search). Participants reported a range of emotions they experienced post-abortion, including relief, sadness, anxiety, guilt, depression, stress, grief, happiness, regret, anger, and a desire to stop or reverse the abortion. The data was aggregated to create an edge list representing the co-occurrence of multiple different emotions. Additionally, women reported any complications experienced and rated nine statements on a 5-point Likert scale regarding their perceptions of being informed during their medication abortion experience.

The statements formed a reliable scale (α = .82), with mean 4.36 (*SD* 0.62). The questionnaire also assessed whether participants needed a follow-up appointment, received or felt the need for a post-abortion ultrasound, sought in-person medical care after the abortion, and identified their sources of medical care and support during and after the abortion.

A single numerical overall score for the perception of being informed was calculated by summing the responses of the nine Likert scale items, with mean 39.22 (*SD* 5.53). Multi-option questions, including those about emotions, were recoded into binary variables (yes/no) for each response item, allowing for the analysis of the individual effect of each response item. Linear regression was then applied to assess the associations between the recoded variables and the perception of being informed.

The co-occurrence data were analyzed using Gephi software to create a network graph. Emotions were represented as nodes, and the co-occurrence of emotions was represented as edges between nodes.

The network was visualized using the Fruchterman-Reingold layout, which positions nodes based on their connections. The thickness of the edges was proportional to the frequency of co-occurrence, with thicker edges indicating more frequent co-occurrences. To identify distinct clusters of emotions, we applied the modularity class algorithm with a resolution parameter of 0.9.

## Results

### Phase I: qualitative findings

Thematic analysis led to four key themes: 1) Seeking information and advice on abortion symptoms and side effects; 2) Seeking help and support for emotional response to abortion; 3) Requesting urgent support and reassurance about the medication abortion; 4) Discussing information provision as part of the medication abortion process.

Below, we describe each theme and corresponding sub-themes with examples and descriptive evidence. For each illustrative quote, we report the source platform and a post identifier (e.g., ‘Netmums post #…’ or ‘Reddit post #…’) to support transparency while minimizing re-identification risk. In accordance with ethical guidance for internet-based research, usernames/handles and timestamps were not retained in the manuscript. Table 2 summarizes the primary qualitative themes, associated subthemes, and illustrative quotes derived from the thematic analysis.. All illustrative quotes are verbatim.

**Table 2:** Initial themes and associated subthemes.

Themes	Associated subthemes	Illustrative quotes
**Seeking information and advice on abortion symptoms and side effects**		
	Pain	“Hi girls. I completed my abortion today at 6 weeks. I’m still having pains in my stomach like radiating round my stomach is this normal? Xx” (Netmums post #3)
	Bleeding	“I’ve been googling for weeks and have read that bleeding should stop after 2 weeks. Except mine hasn’t. The bleeding is less to the point that sometimes it’s just when I wipe. I’m not in any pain and not had any follow ups. Mainly because I didn’t know you were supposed to. I also never took another pregnancy test as I wasn’t told to.” (Netmums post #1)
	Failed abortion	“I had a medical abortion 4 weeks ago. [...] A week after my procedure I became sick. I had a 103/4 degree fever and could barely get out of bed. The clinic gave me some antibiotics and said everything was fine. Fast forward to now, I’m still having pregnancy symptoms. [...] I was wondering if this is normal? And if anyone has had an incomplete abortion? Was it similar to this? Or even a failed one? Thanks in advance.” (Netmums post #21)
	Desiring abortion pill reversal	“I went to start to process of my abortion today […] had the first abortion tablet and I’ve done nothing but regret it and cry and feel so upset by it that I’ve made the worst mistake. I tried 2 hours later to empty my stomach and make myself sick in hope it would help, I’ve spoke to the nurse and all she said is I don’t have to take th second tablets but I’d have to wait for a scan later this week and check if their is astill a heart beat 😟 has anyone had a similar experience? I’m really confused and emotional over this situation is it too late?” (Netmums post #132)
**Seeking help and support for emotional response to abortion**		
	Depression	“I had an abortion in december 2021 and I’m really struggling with coping with it. [...] It’s been nearly 4 months now and I’m worse than ever. I’m depressed all the time and cry every day. I don’t know if I regret my decision but I still feel heartbroken for my baby. I was only 8 weeks, so people supporting me told me not to think of it as a child as it wasn’t properly developed or alive. I felt like it was alive to me and I had a connection with it. I feel like it would have been a boy. I’ve tried counseling but it hasn’t helped and thought maybe chatting to people online could be good for me.” (Netmums post #98)
	Regret	“now 3 months later and it’s the biggest regret of my life I havnt wanted to leave my house, speak to anyone or do anything in that matter whenever I see anything baby related I cry and cry, deap down I know it was for the best but also I just wished I believed in myself more I brought up a beautiful baby girl myself and I could do it again I shouldn’t need to depend on anyone else to do so, I just wish I could go back, how has anyone manage to get over this and start moving on” (Netmums post #89)
	Guilt	“I feel horrible, physically and mentally. I know I did the right thing and that this was the best choice for me, but I never imagined I would feel this much sadness and guilt.” (Reddit post #14)
	Grief	“I didn’t expect the grief. It’s overwhelmed me, but it’s still fairly new.” (Reddit post #9)
	Anxiety	“I’m not scared about the pain nor what’s gonna happen to me all I can think is what a monster I am. Killing something innocent and pure. I’ve never been so depressed in my life. I feel like I’m going insain.” (Netmums post #118)
	Mixed emotions	“I felt relieved and anxious and sad(??) to get the medication. Maybe some part of me was actually becoming attached to this pregnancy.” (Reddit post #35)
**Requesting urgent support and reassurance about the medication abortion**		
	Seeking reassurance	“I just took it a few hours ago. I’m really struggling with it. Any tips on how to make the second pill better? How to not feel like complete and utter shit?” (Reddit post #18)
	Seeking urgent support	“Please, if anyone has had this experience, please lmk. I need help. I already went to the ER and they just told me to let the clots pass through.” (Reddit post #17)
**Discussing information provision as part of the medication abortion process**		
	The consultation	“The consultation consisted of a lot of receiving Ohio mandated texts about other prenatal options which I returned unviewed, waiting, blood work, an ultrasound where I was given the option to look at the screen (I didn’t), they told me that a heartbeat was detected, and an option to keep a print out of the ultrasound (I accepted).” (Reddit post #10)
	Information provision vs. consent	“[I] waited maybe 20-30 minutes, giving me just enough time to read through all the info packets they gave me. You are not allowed to have someone with you, and I believe this is because they don’t want patients to feel forced/ they want this to be 100% your decision. I was put in one room, where the girl asked me a handful of questions. I don’t remember all of them but she reiterated “Do you understand at any point you can change your mind up until the first pill?” and “Do you want to see the ultrasound?” (Reddit post #9)
		“I wasn’t provided as much support as I would have liked, nor was I properly informed of the impact the abortion would have on my body.” (Media article #1)

### Seeking information and advice on abortion symptoms and side effects

Abortion symptoms and side effects for which women most frequently sought to gather information included pain, bleeding, failed or incomplete abortion, and desiring abortion pill reversal.

Experiencing pain and bleeding seemed a point of confusion for many women as they were unsure whether their experience was “normal” when it was just “spotting bleeding and light cramping” as they expected “loads of blood and pain” (Netmums post #35) or when the pain or bleeding was abnormal or too strong:

Today at work I started having rolling waves of cramps getting more and more extreme. I told my husband I felt like I was having contractions and the pain was almost unbearable but I was trying to keep it together at work. I came home sat on the toliet to pee and this thing slipped out of me. Is this a fetus? Left over tissue? Should I got to the ER? (Netmums post #6)

Women also worried if pain and bleeding were suggestive of additional complications, such as infection, as they observed themselves to “randomly bleed nearly every second day and stop again” and asked questions like if “anyone knows why? I don’t think I have an infection as I feel fine” (Netmums post #13).

Some women included detailed descriptions of what they perceived as signs and symptoms of a failed or incomplete abortion and sometimes pictures “(medical abortion) have I passed tissue or the fetus? ×××trigger alert picture×” (Netmums post #77)

Trying to convey the sense of urgency in seeking help and advice, women often included the words “failed abortion” and “help” in the thread title: “Help…not sure if my abortion failed or not 8 weeks” (Netmums post #24); “Failed medical abortion?” (Netmums post #22).

In some cases, women expressed utter shock as a failed abortion was confirmed:

My daughter just went to her Ob dictor today to find out she still pg. […] And after the ultrasound today and we saw a growing baby with a heartbeat and to get told its a little girl we called the place again only to be told the supervisor would return our call .. Guess what still no call... We are floored … And are lost at what to do. (Netmums post #40)

Women often reached out to forum participants for immediate help when they desired abortion pill reversal. They included a call for help in the title of their posts in an apparent effort to get urgent attention: “Second pill taken too late?” (Netmums post #131); “I don’t want to go through with my abortion” (Netmums post #129). Some women expressed immediate regret after taking mifepristone and turned to the Internet to find information on what to do to reverse or stop the process:

I tried 2 hours later to empty my stomach and make myself sick in hope it would help […] has anyone had a similar experience? I’m really confused and emotional over this situation is it too late? (Netmums post #132)

In addition to seeking advice in online forums, some women also reported searching for information by “googling for weeks” and reading “medical journal articles” (Netmums post #1).

Some of the women expressed a desire for professional help and stated they were “angry that I couldn’t go through this with my regular OB, who I know and trust and who knows all my medical history” (Media article #4).

### Seeking help and support for emotional response to abortion

Women sought help from discussion forum participants about their mental and emotional health after completing the medication abortion. The most prevalent emotions mentioned by women were regret, guilt, grief, anxiety, and depression. Some women also reported feeling gratitude and relief.

I had my abortion about 4 weeks ago while I was unsure whether it was really what I wanted...I’ve been very stressed, guilty, angry with my boyfriend, sad, regret. I’m not ok😔. Anyone feeling the same? (Netmums post #104)

Regret, both immediate and delayed (such as several months after the medication abortion), was one of the most frequently noted emotions:

I took my first pill and I was fine. I was fine all night too. Until the next day and the day after when I absolutely broke down again regretting what I had done. […] The feeling of the clots coming out of me while sitting on the toilet for an hour. The brutal pain while the main part was happening. I wish I could go back and change everything. I’m miserable and I can’t stop crying. I feel suicidal. (Netmums post #99)

It’s been 3 months since I went through with a medical abortion. Please tell me this gets easier because I’m really struggling now. (Netmums post #123)

Regret was also expressed as a “wish I could go back” (Netmums post #89) and statements expressing that “I want my baby back” (Netmums #115). Even when women noted that abortion was the right decision, they reported feeling “very emotional, regretful, but knew I’d made the right decision” (Netmums post #22):

I felt so numb, I couldn’t even cry. I spoke to the Mental Health Professional at the hospital and he offered me some numbers for counselling services which I’m waiting for in the mail currently. Physically I was okay and allowed to leave after about 6 hours but mentally I still feel as I did before. All I can think about is being with my poor baby who is all alone and scared and how I should never have let them go. I still have the Pregnancy+ App downloaded on my phone and check for updates and progress, I still make baby names lists and I sleep clutching the pregnancy test which is the only physical reminder I have of my baby. I am completely broken and cannot imagine a future. I received acceptance onto a college course but I felt nothing, I don’t want that life, I just want my baby back. (Netmums post #116)

Some women noted that they regretted choosing abortion while they felt their judgment was clouded, with one woman explaining that she took “those pills in desperation” and that “[t]he world looked so dark to me that I couldn’t imagine bringing a baby into it” (Netmums post #50):

I feel like I made the decision in such a fog of pain. That I wasn’t thinking straight and just wanted to feel like me again, I wanted my life back and to be able to parent my two children […] I don’t know how to get past this. 😢 I keep having flashbacks and seeing the scan and the heartbeat. I want my baby back 😔 (Netmums post #115)

Women also expressed feelings of helplessness, uncertainty, indecision, and a lack of understanding about how to process their current emotions:

…struggling to cope with my emotions. I was referred to a maternity hospital for further treatment which did not help as it was mainly pregnant women and babies in every department and ward which made me feel guilty and also sad that it wouldn’t be me leaving hospital with a baby in a few weeks time. (Netmums post #78)

Notably, women tried to use the discussion forums to ask other women about coping mechanisms and when and how they handled these different emotions. For example, if they were “allowed to […] grieve? Even tho it’s my choice? I want to grieve but is that wrong?” (Netmums post #64):

“It’s only really now I’m realising whats happened and I’m almost grieving but when I do I feel guilty as it was my choice to have the termination in the first place. My boyfriend and I have been having arguments more frequently, which are mainly caused by me as I don’t feel as if he truly understands what I went through. He was extremely supportive and came to all the appointments with me but I just feel so isolated and alone as none of my friends have been through anything similar...I guess I’m just looking for someone else that can understand what I’m going through now to reassure me and talk to.” (Netmums post #78).

Some women noted unexpected emotional pain: “The anticipation and the sadness I felt about it was worse than the pain, which was much more mild than I had anticipated” (Reddit post #20).

This unexpected emotional pain and trauma was exacerbated if women happened to see the fetal tissue:

I started to bleed off the medication and pass clots what was expected. I ended up seeing the fetus witch has totally broke me. I didn’t expect to see it’s little tiny toes and hands perfectly formed. It’s completely made me feel rock bottom. (Netmums post #11)

Sometimes women shared experiences of being “thankful I was able to have this done” (Reddit post #9) and experiencing a sense of relief that they were no longer pregnant. However, often emotions with a positive valence were accompanied by emotions with a more negative valence such as guilt or sadness:

I had a medical abortion in April 2021 and have been struggling with guilt and shame ever since. This is especially when I see pregnant women, particularly those that have other children […] I know the decision for an abortion was right but I really struggle with the guilt. (Netmums post #102)

### Requesting urgent support and reassurance about medication abortion

We observed a sense of urgency in some of the posts, most notably regarding decisions about medication abortion that required swift action. For example, women sought support as they encountered symptoms of a possible failed or incomplete abortion (Netmums post #36) or did not know “what to do” (Netmums post #70).

Women also sought reassurance from the community as an attempt to experience inner peace:

I’m looking for reassurance that this won’t be as horrific as my anxious brain is telling me it will be. (Netmums post #76)

Notably, women often reported a lack of support, feeling “Alone and scared” (Netmums post #80) having “nobody at all to talk to so hoping someone might be able to help🤞” (Netmums post #65), or fearing that the “the judgemental sides will come out” if they shared it with their family (Netmums post #75), which drove them to seek support and reassurance in the online discussion forum:

I sit here guilty and alone as I can not share with anyone who understands. I’m looking for information and support from others who have had a similar experience. (Netmums post #50)

Me and my partner has chosen just to keep it between me and him so I don’t have a lot of support. Here’s my partner thinking I’ll be back to my normal self in a few days and he’s off to football at the weekend but feels like I’ve got on one when I’m feeling like this. And basically I’ve got to get over it with no help. (Netmums post #11)

Women also sought support and reassurance from others online as they shared their experiences with having a lack of decisional certainty and an inability to take the pills, with comments such as “[The] decision is too big. I can’t do it. I’m not strong enough” (Netmums post #128). Another commenter described feeling “numb” and “feel[ing] the whole what if scenario going round in my head and I just feel In limbo” (Netmums post #114).

To express how important it was to receive the support and reassurance via online forums, women returned to forums to pay back and offer their own experience-based advice, support, and encouragement to other women like them:

I thought I’d share my story, as reading experiences here on twox was the only thing that eased my anxieties. (Reddit post #27)

### Discussing information provision as part of the medication abortion process

This theme developed primarily from a subset of data from online magazines, such as *Vice* and *Cosmopolitan*. In these articles, women were asked what it was like to go through the medication abortion process. Their responses included reports of how they received information. Women described information they received about the medication abortion process, the mode by which they received it (e.g., online vs. in-person), and from whom they received it (e.g., medical provider, online, friend). Based on these accounts, we delineate different levels of information provision: consultation, obtaining patient informed consent, and complying with legal requirements to receive abortion.

There was a notable variance across women’s narratives about the consultation process. Some women described going to a clinic and receiving pamphlets, handouts, or watching short informational videos as part of the consultation process. “[T]he required informational video played in a loop in the waiting room,” one woman recalled (Reddit post #10).

Women who did attend a medical facility like Planned Parenthood often relayed that the medical provider took vitals, explained the procedure, and had them sign forms. The types of information received, whether there was a waiting period, or whether an ultrasound was required, often depended upon state mandates:

In [my state] you have to do abortion counseling, so that appointment was like $100. Then you have to make a second appointment to do the procedure or get the pill, and that was going to be around $500. (Media article #6)

Another woman provided a detailed outline of her medication abortion consultation:

I was taken to see a nurse who helped me fill out some forms and explained more of the procedure, how to take the pills, what normal bleeding looked like, when to call the 24-hour Planned Parenthood hotline, and when to go to the ER. (Media article #4)

However, other women talked about desiring to have the medication abortion at home in a private space. Those women who desired to minimize time spent in a medical facility often sought telehealth consultations and medication-by-mail services. In such examples, the informed consent provision was not mentioned in their online narratives. For example, one woman stated:

It felt like an oddly illicit and old-school option to resort to, considering I was a well-insured, internet-savvy person living in New York City—but it was the only way to truly minimize the time spent inside a medical facility. I received my pills without incident, did a telehealth consultation to go over the details, and that was it. (Media article #2)

Women described receiving information from a medical professional as part of a consultation that was typically done face-to-face in a medical facility such as Planned Parenthood. In one report on the experiences of three women who received medication abortion, one woman described how after having a vaginal ultrasound and watching a video about the procedure, “a physician’s assistant came in and went over everything with me one last time and asked if I had any questions.” Finally, she was given a mifepristone pill and advised that after 20 minutes if she “didn’t throw up” the pill would work (Media article #4).

Another woman shared how her medication abortion involved signing a document to ensure “that you understand what the procedure is, that no one can force you to do it, and that you can change your mind at any point until you take that first pill” (Reddit post #9).

Only after she had signed the document, “the actual doctor came in. She wrote up all my prescriptions, and pretty much assessed me on the information to make sure I actually knew what I was doing before giving me the medicine” (Reddit post #9).

Sometimes, women were required to attend multiple appointments that involved varying levels of information received and signed consent forms. One woman provided a descriptive account of her experience:

They gave me a lot of stuff to read about the medications I was going to take. Then they scheduled me for a second appointment the next week. Waiting a whole week was horrible. I couldn’t eat or sleep and was miserable the whole time. I wished they could have given me an appointment the next day. When I finally had my second appointment, I had to fill out some more paperwork and then they took my blood pressure and told me the doctor would give me the medication. […] At the second appointment I didn’t have to wait long. The doctor talked to me about my decision again and then told me I will take the Mifepristone now and then Misoprostol at home 24 hours later. (Media article #5)

Some women desired to have a self-managed abortion, where a woman self-assesses, obtains pills, and conducts the medical abortion without the assistance of a medical provider. Often, these women attempt this type of medication abortion in order to navigate U.S. Food and Drug Administration regulations and any complications or delays required by state mandates. Women who received abortion medication through the mail reported complying with some of the basic requirements (ultrasound and photo ID) but obviously had no physical exam, such as ultrasound or vitals. This type of abortion provision involves a drastically different experience with information provision compared to the experience of women who went to a medical facility, such as Planned Parenthood. One woman described the medication-by-mail process as follows:

they got back to me less than 24 hours later. I sent them a picture of my ultrasound because they wanted that, and they also asked for my photo ID and my address, to send the pills. That was kind of scary because I knew this wasn’t 100 percent legal. But I sent everything in an email, paid the $90 for the pills, and then sent the prescription to the pharmacy in India. (Media article #6)

Notably, some women did not express a need or desire for fully informed decision making. We identified one discussion where a woman tried to communicate the mental and emotional costs of abortion, and the discussion became contentious. In this discussion, women argued whether the commenter’s warnings that “the pain felt during the termination itself is nothing compared to the mental pain and heartache afterwards” (Netmums post #59) was helpful for women who planned to undergo medication abortion. One poster argued that “some of the comments trying to talk you out of this” were “disgusting” (Netmums post #59) while another defended her full transparency:

“The only way to make an informed decision is to be in full possession of ALL the facts. How is keeping other people’s valuable experiences shushed, remotely helpful?! It’s not kind or helpful to the OP to sugarcoat the reality!” (Netmums post #59)

### Phase II: Survey results

#### Sample characteristics.

The study sample consisted of 362 women, with ages ranging between 23 and 54, and a mean of 34.20 (SD 8.15). Most of them identified as White (191, 52.8%) or Black/African American (103, 28.4%), with smaller percentages identifying as Asian (33, 9.1%) or multiple ethnicities (28, 7.7%). Most participants were well-educated, with 45.3% (164) holding a bachelor’s degree and 18.0% (65) a master’s degree. Most women were employed (314, 87.0%), and the most common religious affiliation was Christian (141, 39.1%), followed by Agnostic (107, 29.6%) and Atheist (57, 15.8%). Participants resided in various states, with the highest representation from California (39, 10.8%), followed by Florida (30, 8.3%) and New York (28, 7.7%). The income distribution was diverse, with the largest group of participants (118, 32.6%) reporting an income between $50,000 and $89,000.

In terms of marital status, 40.1% (145) were single at the time of the abortion, and the same proportion (145, 40.1%) were married at the time of data collection. Almost half of the participants had not given birth previously (169, 46.7%), and most had not experienced prior stillbirths (332, 91.7%) or ectopic pregnancies (341, 94.2%). Participant demographic and reproductive characteristics are summarized in Table 3.

**Table 3:** Demographic and reproductive characteristics of study participants.

	Count	%
**Ethnicity**		
Hispanic		
No	48	13.3%
Yes	311	85.9%
Prefer not to answer	3	0.8%
missing	0	
Ages		
23-29	130	35.9%
30-39	150	41.4%
40-54	82	22.7%
missing	0	
**Race**		
White	191	52.8%
Black / African American	103	28.5%
Asian	33	9.1%
Multiple	28	7.7%
Prefer not to answer	5	1.4%
American Indian or Alaskan native	1	< 1%
Native Hawaiian or Pacific Islander	1	< 1%
missing	0	
**Education**		
High school graduate or GED	28	7.7%
Some college or associate degree	92	25.4%
BA	164	45.3%
MA	65	18.0%
PhD / MD	13	3.6%
missing	0	
**Employment status**		
Employed	314	86.7%
Not employed	47	13.0%
missing	1	
**Income**		
0 - 19,000	16	4.4%
20,000 - 49,000	81	22.4%
50,000 - 89,000	118	32.6%
90,000 - 129,000	68	18.8%
130,000 - 149,000	25	6.9%
150,000+	50	13.8%
Prefer not to answer	4	1.1%
missing	0	
**Religion**		
Christian	141	39.0%
Agnostic	107	29.6%
Atheist	57	15.7%
Muslim	8	2.2%
Buddhist	5	1.4%
Jewish	3	0.8%
Hindu	4	1.1%
other	36	9.9%
missing	1	
**Marital status at the time of abortion?**		
Single	145	40.1%
Single but cohabitating	119	32.9%
Married	92	25.4%
Divorced	6	1.7%
missing	0	
**Current marital status**		
Married	145	40.1%
Single	116	32.0%
Single but cohabitating	86	23.8%
Divorced	13	3.6%
missing	2	
**First experience of medication abortion?**		
Yes	343	94.8%
No	19	5.2%
missing	0	
**Pregnancy confirmation**		
Both pregnancy test and ultrasound	208	57.5%
Pregnancy test	127	35.1%
Ultrasound	22	6.1%
Other	4	1.1%
missing	1	

### Survey results regarding informed consent

Among the 362 women surveyed, most of them (235, 64.9%) obtained a medication abortion from an in-state abortion clinic, with an additional 8.6% (31) receiving care from an out-of-state clinic. A smaller percentage of women received the medication abortion through their regular doctor’s office, either in person (38, 10.5%) or via telehealth (2, 0.5%). Online orders accounted for 9.4% (34) of abortions, with varying pick-up locations noted by the women. Overall, the results demonstrate a preference for clinic-based care, with a small but significant portion of women utilizing telehealth or online options.

A plurality of participants (42, 11.6%) obtained their abortion in California, followed by New York (29, 8.0%) and Florida (26, 7.2%). Other frequently reported states included Georgia (20, 5.6%), North Carolina (20, 5.5%), and Virginia (17, 4.7%).

Most participants (353, 97.5%) reported that their abortion provider enabled them to give informed consent prior to starting the abortion process. This process was primarily conducted in person (313, 86.5%), with some participants also receiving information via phone calls (8, 2.21%), video calls (14, 3.9%), websites, or mailed materials. Notably, 11.0% (40) did not provide informed consent in person. Most women provided informed consent in writing (202, 55.8%), while others did so verbally (143, 39.5%). The medical provider who prescribed the abortion pills was the primary person involved in the informed consent process (197, 54.4%). While the level of acquired information provided was generally high, with a mean score of 4.36 out of 5, those who experienced complications during the abortion (101, 27.9%) reported a significantly lower perception of being informed (Mann-Whitney U = 9084.00, *P* < .001, Δ _Med_ = 0.22, Δ _SE_ = 0.03). However, most participants (342, 94.5%) did not desire additional information beyond what was provided.

In response to the open-ended question “Did you desire to receive more information about the medication abortion process than what was offered? (If yes, please explain what additional information you would have liked to receive.),” women noted the need for more information about long-term effects, mental health care and services, counseling, coping post-abortion, expected level of pain and trauma, risk of infection, pros and cons of medical versus surgical abortion, available options (e.g., “if i wanted to keep the baby/help me change my mind”), and wished that “ultrasound was a part of a follow up after an abortion.”

### Survey results regarding individual experiences

**Fig 2:**
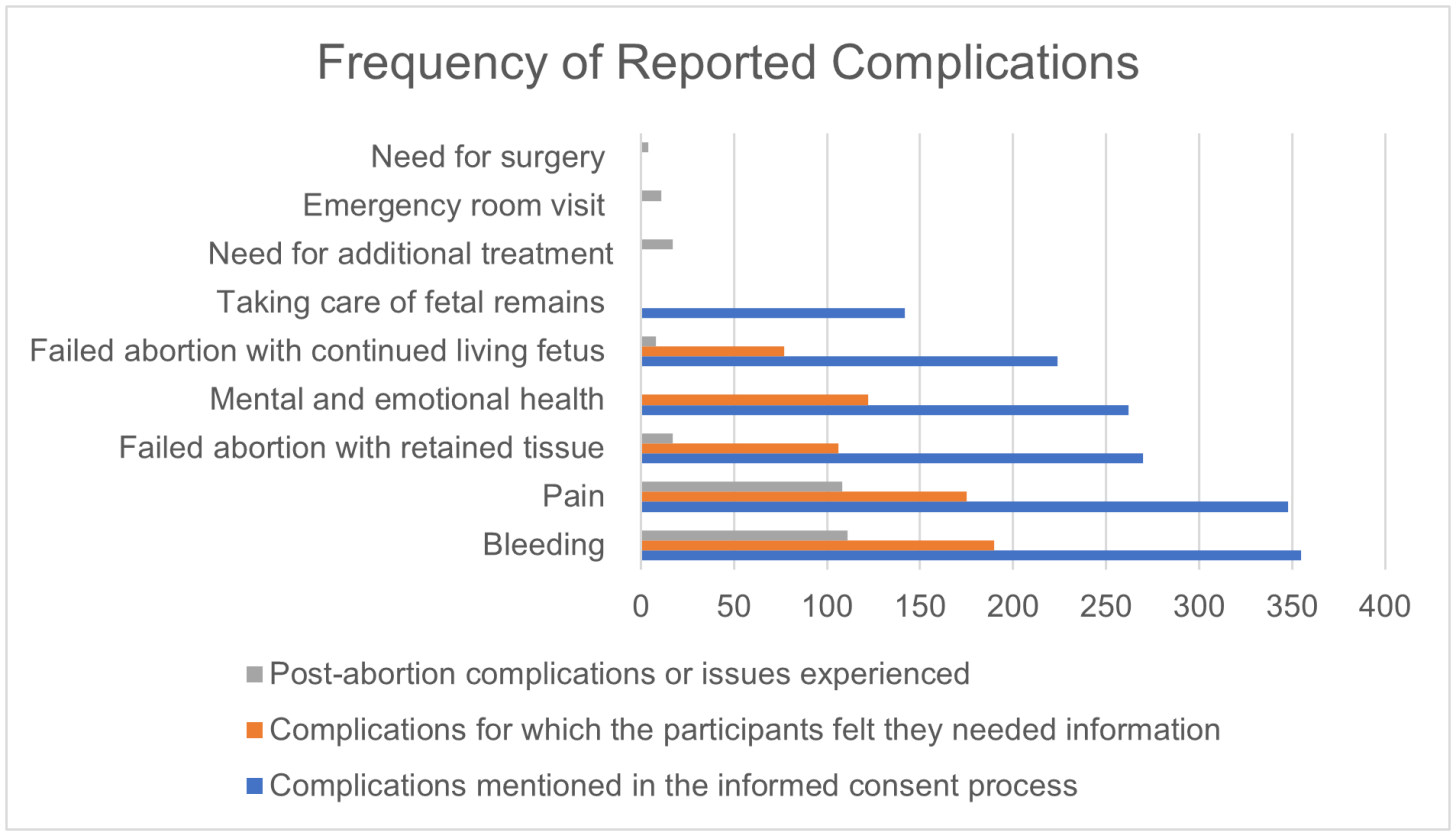
Frequency of reported complications across 3 survey items.

**Possible post-abortion complications mentioned in the informed consent process.** Possible complications most frequently recalled as addressed in the informed consent process included bleeding (355/362, 98.1%), followed by pain (348/362, 96.1%), incomplete abortion with retained tissue (270/362, 74.6%), mental and emotional health (262/362, 72.4%), failed abortion with continued living fetus (224/362, 61.9%), and seeing and taking care of fetal remains (142/362, 39.2%) (Fig 2).

**Complications for which the participants felt they needed more information.** The most frequently cited complications participants felt they needed more information than was provided during the informed consent process included bleeding (190/362, 52.5%), pain (175/362, 48.3%), mental and emotional health (122/362, 33.7%), incomplete abortion with retained tissue (106/362, 29.3%), and failed abortion with continued living fetus (77/362, 21.3%) (Fig 2).

**Post-abortion complications or issues experienced during abortion**. The most frequently mentioned post-abortion complications included unexpected levels of bleeding (111/362, 30.7%) and pain (108/362, 29.8%). Other complications reported were incomplete abortion with retained tissue (17/362, 4.7%), the need for additional treatment (17/362, 4.7%) or an emergency room visit (11/362, 11, 3%), failed abortion with continuing living pregnancy (8/362, 2.2%), and the need for surgery (4/362, 1.1%) (Fig 2).

**Post-abortion follow-up appointment and support.** 47.8% of the participants (173/362) had an in-person follow-up appointment scheduled, and a similar proportion (183/362, 50.5%) felt they needed one. However, half of the 362 women neither received a post-abortion ultrasound (182, 50.3%) nor felt they needed one (213, 58.8%). While only 31.5% (114/362) of women reported seeking medical care after their abortion, 151 (41.7%) indicated that they sought medical care from their provider post-abortion. Women sought support from various sources, including partners, family, friends, and online forums. However, 9.7% (35/362) felt they needed support but did not receive it.

### Survey results regarding emotional responses

**Fig 3:**
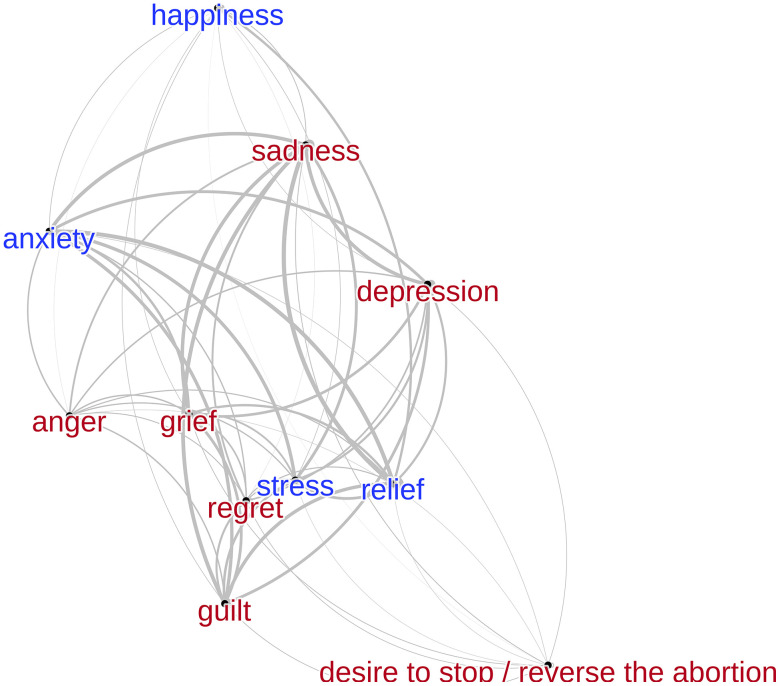
Network of emotional responses associated with unwanted pregnancy and potential abortion (Nodes = 11, Edges = 55). Nodes represent distinct emotions, and edges indicate their co-occurrence in individuals’ experiences. The network is organized into two communities (green and red) detected using the modularity class algorithm (resolution = 0.9), available in the Gephi software, which identifies clusters of emotions that are more densely interconnected within their own group than with the other group.

The emotional responses most frequently reported by participants included relief (248/362, 68.5%), sadness (184/362, 50.8%), anxiety (151/362, 41.7%), guilt (141/362, 39%), depression (122/362, 33.7%), stress (120/362, 33.1%), and grief (110/362, 30.4%). Less frequent emotions included happiness (70/362, 19.3%), regret (63/362, 17.4%), anger (52/362, 14.4%), and a desire to stop or reverse the abortion (23/362, 6.3%). The network analysis generated a graph with 11 distinct emotions (nodes) connected by 55 edges, representing the co-occurrence of emotions (see Fig 3). The thickness of the edges indicates the frequency of co-occurrence, with thicker edges reflecting more frequently reported emotional pairs (the edge list is included in the Appendix). Two main clusters emerged from the analysis, reflecting distinct emotional communities.

**Cluster 1: “Approaching Emotions” (Green Nodes).** This cluster includes relief, anxiety, stress, and happiness. These emotions may reflect attempts at acceptance or coping with the abortion experience. The strongest connections within this cluster are between relief and sadness (111 occurrences), relief and anxiety (102 occurrences), and anxiety and stress (85 occurrences). The presence of relief in this cluster indicates that, despite the overarching relief felt by many women, it co-occurred with significant distress-related emotions (e.g., anxiety and sadness). This co-occurrence suggests that relief may be accompanied by complex, simultaneous emotional experiences. Happiness, while part of this cluster, appears less central with fewer co-occurrences, linking with relief, anxiety, and stress. It is connected to relief, implying that happiness might be associated with positive outcomes for some women, though this is less frequent compared to the distress-based emotions.

**Cluster 2: “Avoidance Emotions” (Red Nodes).** The second cluster is dominated by emotions like guilt, sadness, regret, depression, anger, and grief. These emotions are likely reflective of distress, internal conflict, or emotional struggle regarding abortion. Strong connections within this cluster include guilt and sadness (100 occurrences), grief and sadness (92 occurrences), and depression and guilt (75 occurrences). These connections suggest that guilt and sadness form the emotional core of distress in the medication abortion experience. Sadness is a central node in this network, appearing frequently in connection with other avoidance emotions such as grief, guilt, and depression, signifying its pivotal role in emotional processing after abortion. Regret and anger, though less frequent than other emotions, still show important links with guilt and sadness, reinforcing the pattern of emotional conflict in this group.

**Interconnections Between Clusters.** The analysis shows some notable cross-cluster connections between approaching and avoidance emotions, indicating emotional ambivalence: relief, a positive emotion, is strongly linked with negative emotions such as sadness (111 occurrences), guilt (87 occurrences), and grief (63 occurrences). Anxiety also bridges the two clusters, connecting with sadness (94 occurrences) and guilt (86 occurrences).

### Linear regression results

Table 4 presents the results of a regression analysis based on a hierarchical approach testing the associations between various predictors and women’s perception of being informed. The analysis was conducted in three sequential blocks to explore the associations between various factors and women’s “perception of being informed” during medication abortion: Block 1 includes issues mentioned in the informed consent process; Block 2 adds issues where women felt the greatest need for information; and Block 3 incorporates emotional responses during the abortion process. Changes in *R*² (*ΔR*²) reflect the incremental contribution of each block to the overall model. Significant associations are highlighted, with the final model explaining 21% of the variance in perceived informativeness (*R*² = .21).

**Table 4:** Hierarchical regression analysis of factors associated with women’s perception of being informed during medication abortion.

Model	Predictor	*B*	*β*	*SE*	*95%CI*		*P*	*R*²	*p ΔR*²
					*LI*	*UL*			
Block 1: Issues mentioned in the informed consent process	Intercept	34.83		2.01	30.87	38.79	< .001	.12	
	bleeding	2.75	.08	2.10	−1.38	6.88	.19		
	failed abortion (continued living fetus)	0.18	.02	0.71	−1.21	1.57	.80		
	failed abortion (retained tissue)	1.54	.12	0.82	−0.08	3.16	.06		
	mental and emotional health	1.53	.13	0.67	0.21	2.86	.02		
	pain	0.33	.01	1.49	−2.61	3.27	.83		
	seeing and taking care of fetal remains	2.37	.21	0.63	1.13	3.61	< .001		
Block 2: Issues for which women felt greatest need for information	bleeding	−0.20	−.02	0.64	−1.46	1.06	.76	.15	.03
	failed abortion (continued living fetus)	0.90	.07	0.88	−0.84	2.64	.31		
	failed abortion (retained tissue)	−1.93	−.16	0.80	−3.52	−0.35	.02		
	mental and emotional health	−1.28	−.11	0.64	−2.54	−0.01	.047		
	pain	−0.01	.00	0.65	−1.29	1.27	.98		
Block 3: Emotions experienced during the medication abortion process	anger	0.48	.03	0.90	−1.29	2.25	.60	.21	.02
	anxiety	0.58	.05	0.68	−0.75	1.91	.39		
	depression	−0.49	−.04	0.75	−1.96	0.98	.51		
	desire to stop / reverse the abortion	−2.31	−.10	1.30	−4.86	0.25	.08		
	grief	0.09	.01	0.75	−1.38	1.56	.90		
	guilt	−0.37	−.03	0.70	−1.76	1.02	.60		
	happiness	1.59	.11	0.74	0.13	3.05	.03		
	regret	−0.06	.00	0.97	−1.97	1.85	.95		
	relief	−0.80	−.07	0.67	−2.12	0.53	.24		
	sadness	0.31	.03	0.66	−0.98	1.61	.64		
	stress	−2.29	−.20	0.67	−3.61	−0.97	< .001		

Model 1 started with issues mentioned in the informed consent process, identifying significant associations for “seeing and taking care of fetal remains” (B = 2.37, SE = 0.63, β = .21, *P* < .001) and “mental and emotional health” (B = 1.53, SE = 0.67, β = .13, *P* = .02), yielding an *R*² of .12. Model 2 added a second block focusing on issues where women felt the greatest need for information, which further explained the variance, with significant associations found for “failed abortion (retained tissue)” (B = −1.93, SE = 0.80, β = −.16, *P* = .02) and “mental and emotional health” (B = −1.28, SE = 0.64, β = −.11, *P* = .047). This addition increased the model’s explanatory power to *R*² = .15, with a *ΔR*² of .03. Model 3 incorporated a third block that examined emotions experienced during the medication abortion process, where “stress” (B = −2.29, SE = 0.67, β = −.20, *P* < .001) and “happiness” (B = 1.59, SE = 0.74, β = .11, *P* = .03) were significantly associated with the perception of being informed. The final model had an *R*² of .21, with a *ΔR*² of .02.

The regression analysis revealed how different factors cumulatively contributed to women’s perception of feeling informed during the medication abortion process. The first block, which focused on issues mentioned in the informed consent process, showed that concerns about “seeing and taking care of fetal remains” and “mental and emotional health” were significantly associated with an increase in feeling informed. The second block, which addressed areas where women expressed the greatest need for additional information, highlighted the importance of addressing specific concerns, such as “failed abortion (retained tissue),” and further underscored the role of mental and emotional health in shaping women’s perceptions. The final block, which included emotional responses, revealed that emotions like stress and happiness were also significantly associated with how informed women felt, with stress being associated with a lower perception of being informed and happiness with an increased perception of being informed.

### Integration of quantitative and qualitative findings

We created a joint display of the findings (see Table 5) to highlight the extent to which the quantitative and qualitative results converged or diverged and in what ways [36]. We selected all variables from Phase 2 quantitative results that were significantly associated with women’s perception of being informed and listed them in the column to be compared for congruency/discrepancy with the qualitative themes displayed in rows.

**Table 5:** Joint display of quantitative and qualitative findings.

	Quantitative variables*	Statistical significance	Quantitative and Qualitative congruency	Qualitative themes and quotes
**Issues for which women felt the greatest need for information**	Mental and emotional health	Significant (B = −1.53, 95% CI 0.21, −2.86, *P* = .02)	Congruent	**Seeking help and support for emotional response to abortion** “I was told I would be relieved after it was over, but I feel worse than before....” (Reddit post #14)
	Seeing and taking care of fetal remains	Significant (B = 2.37, 95% CI 1.13–3.61, *P* < .001)	Congruent	**Seeking help and support for emotional response to abortion** “I ended up seeing the fetus witch has totally broke me. I didn’t expect to see it’s little tiny toes and hands perfectly formed. It’s completely made me feel rock bottom.” (Netmums post #11)
	Incomplete abortion (retained tissue)	Significant (B = −1.93, 95% CI −3.52 - −0.35, *P* = .02)	Congruent	**Seeking information and advice on abortion symptoms and side effects** “Help…not sure if my abortion failed or not 8 weeks” (Netmums post #24)
**Emotions**	Stress	Significant (B = −2.29, 95% CI −3.61, −0.97, *P* < .001)	Congruent	**Seeking information and advice on abortion symptoms and side effects** “Please, if anyone has had this experience, please lmk. I need help. I already went to the ER and they just told me to let the clots pass through” (Reddit post #17) **Requesting urgent support and reassurance about medication abortion** “Taking abortion pill alone and secretly because I cannot tell my partner. Please give help and advice I’m so so scared.” (Reddit post #32) “I just needed to type this all down, as I don’t have anyone I can say it all to.” (Netmums post #121).
	Happiness	Significant (B = 1.59, 95%CI 0.13, −3.05, *P* = .03)	Lack of congruency	**Seeking help and support for emotional response to abortion** “I felt relieved and anxious and sad(??) to get the medication. Maybe some part of me was actually becoming attached to this pregnancy.” (Reddit post #35)

We found consistent agreement between quantitative and qualitative data. Specifically, two variables for which women felt the greatest need for information – mental and emotional health and seeing and taking care of fetal remains – were congruent with the qualitative finding that women are seeking help and support for their emotional response to abortion. Incomplete abortion (retained tissue), also included under the issues for which women felt the greatest need for information, was congruent with the theme “seeking information and advice on abortion symptoms and side effects.”

Similarly, we found agreement between the variable stress (from the Emotions category) and two qualitative themes: 1) Seeking information and advice on abortion symptoms and side effects; and 2) Requesting urgent support and reassurance about medication abortion. Qualitative data under these two themes consisted of women’s expressions of being “in shock” (Netmums post #51), “terrified” (Netmums post #63), “stressed” (Netmums post #104), having uncertainty, and requiring reassurance due to high levels of anxiety and stress.

Happiness (Emotions), however, was not represented in the qualitative findings. One potential confounder to this congruence was the way emotion was expressed as “mixed emotions” in our qualitative analysis. Women who expressed a sense of relief and happiness following their medication abortion, simultaneously mentioned a sense of sadness, among other emotions. Next, in terms of frequency, we also found fewer posts mentioning “happiness” or even “mixed emotions.”

To add more clarity to this part of the research, we conducted additional data analysis: network analysis, which showed notable cross-cluster connections between the emotions. Specifically, we observed a strong link between relief and sadness, guilt and grief. These connections suggest that women might experience relief from resolving the pregnancy while still grappling with guilt or sadness about the abortion. Similarly, anxiety was also connected with sadness and guilt. This connection indicates that, even when experiencing relief or coping, women may still feel anxious about the broader implications of the abortion.

We also see a divergence between the qualitative and quantitative findings around information provision. The theme “Discussing information provision as part of the medication abortion process” is more reflective of women’s confusion about what information they should be provided with and by whom, than offering any insights on trusting abortion providers as their primary source of information. As a general sentiment, women appeared to trust doctors most, wished they could undergo abortion with a doctor’s supervision, and placed highest value on the information if it was shared by a doctor. For example, a woman discussing her failed abortion posted this comment:

Yes I’m in shock, I don’t know what to do and have been advised by my doctor that the abnormalities are extremely severe if I were to continue. This is a doctor, not an Abortion clinic. (Netmums post #51).

## Discussion

The objective of this study was to examine women’s perceptions of the information they need to feel adequately informed when consenting to medication abortion. Using an exploratory sequential mixed methods design, we integrated qualitative analysis of online narratives and news stories with quantitative survey data to characterize the types of information women seek, how they interpret their experiences, and how informational and emotional factors relate to perceptions of being informed. Together, these findings provide insight into how informed consent is experienced and interpreted by women navigating medication abortion in contemporary care contexts.

As medication abortions often take place outside a medical setting, with a woman undergoing the process in the privacy of her own home, care is needed to ensure the pre-abortion informed consent appropriately educates her about what she may experience. The decision to have a medication abortion warrants verbal, written, and visual information regarding the proposed intervention and risks, benefits, and alternatives of the available options, including time to consider the potential short and long-term consequences of each option. Across both phases of the study, women most frequently sought information related to physical symptoms and side effects, emotional responses, and reassurance about whether their experiences were expected or indicative of complications. Notably, they often expressed a heightened sense of urgency and need for support as they sought out this information online. Women often communicated feeling lost, confused, unsure, lack of decisional certainty, and a desire to reverse the effect of medication abortion drugs. Bleeding, pain, and mental and emotional health were the most frequently cited issues for which the survey participants felt they needed more information than was provided during the informed consent process. However, survey respondents also cited these same issues as being part of the information shared during the informed consent process, with clinical care guidelines stating that patients should be counseled about the level of pain and bleeding that is expected and when to contact a medical provider [37], with patients at later gestational ages informed of the possibility of seeing pregnancy tissue and recognizable fetal parts [38]. This discrepancy suggests that while women are generally being informed about medication abortion, the information provided may not always meet their desired level of detail or comprehensiveness, contributing to perceptions of uncertainty during decision-making. Our findings highlight the importance of the quality and adequacy of information rather than its presence or absence.

Direct references to informed consent, or information provision by abortion providers, was limited in most online discussions included in our study. In some reports, there was a recognition that legally mandated requirements existed, but what information women expected as part of this process is unclear. However, frequently expressed confusion about the abortion process, risks, and available options; expressions of indecision and regret; and attempts to seek additional information online highlight areas where women experienced uncertainty or sought reassurance regarding the evidence-based risks, benefits, and realities of medication abortion. Providing this information is a critical part of comprehensive informed consent [39,40].

We note similar confusion and lack of understanding of what informed consent entails in the survey results. While most women reported receiving informed consent in person (87%), only about half (58%) of the women reported consenting to the medication abortion in writing. We find this discrepancy unexplainable and can only speculate that the women either misunderstood the question or were unable to recall how they provided informed consent.

Further, while most women in our survey indicated a need for more information regarding certain complications, 95% stated that they did not desire additional information beyond what was provided. This discrepancy mirrors the inconsistency we observed in women’s reports of seeking medical care after their abortion. These sentiments also contrast with qualitative findings showing women seeking information and advice, often urgently, through online discussion forums and search engines, reflecting ongoing information-seeking and reassurance-seeking during the medication abortion process.

A network analysis of emotions revealed that the co-occurrence of relief with negative emotions like sadness, guilt, and grief suggests a nuanced personal emotional experience during and after a medication abortion. Relief may stem from the resolution of an unwanted pregnancy, but it does not preclude the simultaneous presence of regret, sadness, or guilt, which could be tied to personal, social, or moral discourses surrounding abortion. The central role of sadness highlights its importance as a shared experience, appearing frequently across both clusters. The connections of sadness to a wide array of emotions suggest that it may serve as a pivotal emotion in reconciling the abortion experience. Guilt and depression, as seen in their strong links to other emotions with a negative valence (e.g., sadness, regret), highlight the psychological burden some women may carry, indicating that guilt is deeply interwoven with other negative affective states, which may require targeted psychological intervention during a woman’s coping experience. The network’s structure, therefore, paints a picture of emotional ambivalence: women often experience relief alongside distress, navigating a complex emotional landscape that involves both approaching and avoidance strategies. This duality suggests the need for emotional support that addresses not just the initial relief but also the deeper, lingering feelings of sadness, guilt, and regret, that many women simultaneously experience.

Importantly, seeking additional information online should not be interpreted as evidence of inadequate counseling, but rather as a common and multifaceted behavior reflecting reassurance-seeking, cross-checking, emotional processing, or preference for multiple information sources. Individuals commonly seek additional information online as part of routine health decision-making, particularly in contexts involving uncertainty, stigma, or emotional distress [41,42]. In this study, online narratives reflect how women processed and contextualized their experiences rather than serving as a direct measure of the quality or completeness of counseling received. Accordingly, our findings are best understood as identifying areas where women seek clarification or reassurance, rather than as demonstrating systematic deficiencies in informed consent practices.

Informed consent is widely understood not as a singular event but as an ongoing process that requires meaningful disclosure, comprehension, and the opportunity for patients to reflect on information, while considering their values and circumstances [4,6,43,44]. Within this framework, our findings suggest that women’s perceptions of being informed during medication abortion are shaped not only by the availability of information, but also by how well that information prepares them for anticipated physical and emotional experiences. Existing literature supports these findings showing that decision‑making in pregnancy and birth care is dynamic, embodied, and deeply shaped by uncertainty and moral expectations, suggesting that informational adequacy cannot be reduced to completion of consent procedures [45–47].

Systematic reviews of telemedicine, including telemedicine abortion, document generally high satisfaction and safety but highlight enduring concerns around informed consent, quality of communication, and variability in how risks and experiences are explained [48–53]. Research on telemedicine abortion has shown that while most patients report receiving adequate information, gaps may persist in how risks, side effects, and expected experiences are communicated, interpreted, and recalled, particularly with pain, bleeding, emotional responses, and the management of fetal tissue. Our findings align with this literature by suggesting that informational adequacy cannot be evaluated solely by whether consent procedures were completed but must also consider whether information is delivered in ways that match patients’ expectations, emotional states, and informational preferences. The frequent emphasis on uncertainty, reassurance-seeking, and emotional distress observed in both qualitative narratives and survey responses highlights the importance of consent practices that extend beyond factual risk disclosure to include anticipatory guidance and emotional support.

The expansion of telemedicine abortion has introduced new opportunities to increase access, while also altering the context in which informed consent is delivered. In remote care settings, patients may have fewer opportunities for real-time clarification or reassurance, potentially increasing reliance on online sources for supplemental information. Importantly, seeking additional information online does not necessarily indicate inadequate counseling; rather, it may reflect a reasonable desire to verify, personalize, or emotionally process information received during the consent process. Our findings underscore the need for telemedicine models to anticipate this behavior and proactively address common areas of uncertainty.

Within this context, the concept of preference misalignment may serve as a useful interpretive lens rather than a demonstrated outcome of this study. Although we did not directly assess patients’ initial values, deliberative processes, or preference formation, our findings suggest that mismatches between the information emphasized during consent and the issues women later seek information about could create conditions under which preferences are not fully supported. Framed cautiously, this highlights a potential risk rather than a documented failure and underscores the importance of consent processes that are iterative, responsive, and tailored to patient concerns. Research on consent interventions shows that interactive and tailored approaches improve comprehension and values‑congruence, underscoring the need for iterative, preference‑sensitive consent processes rather than standardized, checklist‑driven models [6,43,44,54].

Our study has several important methodological limitations, which should influence the generalizability and applicability of our findings. First, determining the exact geographic location of discussion forum participants was not always possible as there were no direct indications of location. We paid particular attention to the Netmums forum as it is based in the UK and is a primary source of our qualitative phase research, offering rich data. Although we excluded any discussion posts that contained a reference to the UK health system and any linguistic nuance indicating UK characteristics, it is likely that we were unable to identify all posts from non-U.S.-based women and therefore they were included in the analytical dataset. Similarly, Reddit has an international userbase and the nationality of individual posters was usually not obvious. However, we balanced this limitation by including only U.S.-based women in our survey sample. We also acknowledge a smaller sample size of the Phase 2 survey and recognize that some of our findings should be interpreted with caution. However, for our analysis we had sufficient statistical power to make appropriate inferences comparing variable relationships and associations with a sampling error around the estimates of approximately + /-5%.

Representativeness and recruitment bias are important limitations of both phases. The qualitative data reflect narratives shared in public online spaces, which are inherently self-selected; individuals who post about medication abortion may differ from those who do not engage online. Similarly, the Phase II survey relied on recruitment through Prolific, an online research platform, and therefore represents a nonprobability internet sample. Although online panels can efficiently recruit diverse participants, they may underrepresent individuals with limited internet access, lower digital literacy, or greater privacy concerns, and may overrepresent those who are more comfortable disclosing sensitive experiences in an online setting. These factors may limit the generalizability of prevalence estimates; however, the study’s primary aim was to examine patterns of perceptions and associations related to informed consent, and the convergence of findings across qualitative and quantitative phases strengthens confidence in the salience of the themes identified.

In addition, as the qualitative and quantitative samples were different, the integrated results cannot directly explain the congruency. For example, in our findings where we report a lack of congruency for happiness, it is possible that women who reported these emotions in the survey are not posting on discussion forums. However, despite these samples being independent, there is a broader applicability to the experiences of women in general and their emotional reactions to medication abortion. Further, the insights gained from the sequential approach to analyzing two different analytical datasets allowed a purposeful data generation, while integration of the qualitative and quantitative findings enabled a deeper understanding of women’s experiences during medication abortion. Very importantly, our research offers an analysis of women’s voices that may often be stifled in tense, politically, and ideologically driven debates surrounding medication abortion.

Additional limitations include possible recall bias in both the forum posts and survey responses, as participants may not accurately remember or may reinterpret the information received during their abortion experience. Self-selection bias may also have influenced our data, as women who chose to post online or respond to our survey may differ systematically from those who did not. Furthermore, the influence of abortion-related stigma may have shaped how women narrated or withheld certain details about their experience, particularly concerning informed consent and emotional responses.

We also acknowledge that our analysis did not differentiate between two distinct models of medication abortion: regulated telehealth abortion and unregulated online pharmacy abortion. Regulated telehealth abortion involves care delivered by licensed healthcare professionals through established telemedicine platforms that adhere to state and federal guidelines. These services may include comprehensive components such as patient screening, counseling, informed consent, and follow-up care, although the degree of counseling or follow-up varies depending on the service. In contrast, unregulated online pharmacy abortion refers to the purchase of abortion medications from online sources that may operate outside of formal medical or legal oversight. These sources often lack provider consultation, standardized informational materials, and safety protocols, which raises concerns about quality, safety, and informed decision-making. Although these two models can differ significantly in terms of structure and oversight, our analysis did not identify clear distinctions in how women described receiving information based on the care model they used. Neither the personal narratives nor the survey responses contained explicit references to different informed consent processes, levels of counseling, or follow-up support that could be linked to the regulatory status of the abortion model. This absence may reflect limitations in women’s awareness of the type of care they received, a lack of transparency in service delivery, or a general consistency in informational gaps across models. Further research is needed to investigate how the care model, regulated or unregulated, influences the quality and content of informed consent and overall patient experience.

This research provides insight into women’s experiences in a way that is both consequential and illustrative, particularly in how women’s emotional experience is consistent in its high variability and how women express a clear desire or need for more access to abortion-related information. These findings are consistent with other reports of “global demand” for information [55]. Our finding that experiencing adverse symptoms or complications (most commonly pain and bleeding) was correlated with feeling uninformed is also consistent with other research indicating that the experience of more pain and bleeding than expected is a predictor of not choosing medication abortion again [56]. One study found that a common framing of the pain associated with medication abortion, likening symptoms to those resulting from a bad period, can cause some women to underestimate the severity of the pain they will encounter and lead to poor and frightening experiences during the medication abortion process [57]. Thus, properly counseling women about pain and bleeding could result in fewer perceived complications and greater perceptions of feeling informed.

Our findings contrast with other research suggesting high decisional certainty among women who decided to end their pregnancy [58]. We see a need to be more nuanced in assessing decisional certainty. Following this approach, for women who are certain of their decisions, abortion providers may provide clinical assessment and discussion of pros and cons for medication versus surgical abortion [39]. For patients who are unsure, providers should offer a nonbiased discussion of options, including parenting or adoption, alongside options for abortion [39].

In many cases these are complex decisions that involve institutional and legal considerations which may affect timing and availability of options. Care providers should offer accurate, evidence-based, and individualized counseling. Clinical assessment, informed consent, and patient education are essential components in evaluating a patient before abortion.

Tools to support information sharing such as patient-centered decision aids, which are widely available for many clinical areas, are lacking for abortion provision. A qualitative content analysis of 49 patient decision aids comparing surgical and medication abortion found that the information presented in the decision aids was inconsistent and sometimes biased [59]. Recent research describing the development of a decision aid, adapted to a Canadian context, demonstrated the ability to meet a need to support informed choices about abortion method that were consistent with women’s values [60]. Developing such tools for the provision of abortion should become a priority to ensure that women’s preferences in abortion decision-making are adequately explored and respected. Providing a medication abortion without proper counseling can lead to misdiagnosis of preferences [61]. Women’s reports in our qualitative phase indicate that this is happening more than is often acknowledged. In addition to a need for better understanding of the factors that influence women’s preferences for medication abortion, as noted in the existing research [62], our research suggests that there is a massive gap in understanding women’s needs and preferences for information provision and counseling for medication abortion.

## Conclusions

The findings from this research underscore the critical role of comprehensive information provision in the context of medication abortion. Healthcare providers should seek ways to enhance women’s informed consent for medication abortion. Informed consent for medication abortion is a complex and multifaceted process that requires careful consideration of patients’ social support networks, information needs, and decision-making preferences. Future research should continue to explore innovative approaches to informed consent and the development of decision aids that address the diverse needs of patients, especially in the context of changing abortion provision where women often experience increased vulnerability and uncertainty.
